# PPAR-γ, RXR-α, and VDR Expression in Gingival Tissues of Patients with Grade B and Grade C Periodontitis: A Cross-Sectional Clinical Immunohistochemistry Study

**DOI:** 10.3390/jcm15082957

**Published:** 2026-04-13

**Authors:** Ozkan Karatas, Fikret Gevrek

**Affiliations:** 1 Department of Periodontology, Faculty of Dentistry, Tokat Gaziosmanpasa University, 60250 Tokat, Turkey; 2 Department of Histology and Embryology, Faculty of Medicine, Tokat Gaziosmanpasa University, 60250 Tokat, Turkey

**Keywords:** immunohistochemistry, nuclear receptors, periodontitis

## Abstract

**Background/Objectives:** Periodontitis grade reflects differences in disease progression and risk, yet the underlying host-response signatures that distinguish grade B from grade C are not fully elucidated. Nuclear receptors involved in inflammation and tissue homeostasis may contribute to these biological differences. The present study aimed to evaluate the expression of peroxisome proliferator-activated receptor-γ (PPAR-γ), retinoid X receptor-α (RXR-α), and vitamin D receptor (VDR) in gingival tissues from periodontally healthy individuals and from patients with grade B and grade C periodontitis, with the primary comparison focusing on grade-related differences within the same disease stage (stage 3). **Methods:** Forty-five participants were allocated to three groups: Group 1, healthy controls; Group 2, stage 3 grade B periodontitis; and Group 3, stage 3 grade C periodontitis. Clinical parameters, including plaque index (PI), gingival index (GI), and clinical attachment loss (CAL), were recorded. Fibroblast and inflammatory cell density, and immunohistochemical expression levels of PPAR-γ, RXR-α, and VDR were assessed on histological sections. **Results:** Compared with healthy controls, both periodontitis groups showed lower fibroblast cell counts and higher inflammatory cell counts. PPAR-γ expression was significantly higher in Group 3 than in the other groups, whereas RXR-α and VDR expression were higher in Group 1 than in Groups 2 and 3. **Conclusions:** These findings suggest that increasing disease grade within stage 3 periodontitis is associated with increased PPAR-γ expression, whereas RXR-α and VDR expression primarily distinguish healthy from diseased gingival tissues. This nuclear receptor profile may help explain biological differences between healthy, grade B and grade C periodontitis and support future risk-stratified host-modulatory approaches.

## 1. Introduction

Periodontitis is a chronic, destructive disease of the periodontal tissues, which is initiated by the dysbiotic oral microbiota and progresses with the host’s immune response against this microbiota. Several factors, such as systemic, genetic, or environmental factors, modify the course of the disease [1,2]. The disease development and progress depend on the humoral and innate immune response, which includes T cell activation, host-derived proteinases, inflammatory pathways, and nuclear receptor activations [3]. Inflammation starts in the marginal gingival epithelium and progresses to the periodontal ligament and alveolar bone [2,4]. The severity and extent of the inflammation, complexity, future risk, and systemic effects of periodontitis differ with the diagnosis, which is categorized by stage and grade of involvement [2,4]. Furthermore, the etiopathogenesis of the disease in terms of the role of different receptor activations in the disease development and progression has not been clarified yet.

There are several pathways in the development process of periodontitis. The major one is the nuclear factor-κB (NF-κB), which regulates inflammatory and destructive processes in the periodontal tissues [3]. Inflammation and consequent bone loss in periodontitis are associated with increased osteoclastic activity and impaired osteoblastic function [5,6]. NF-κB and receptor activator of nuclear factor kappa-B ligand (RANKL) are key regulators of osteoclast proliferation, differentiation, and activation in inflammatory bone loss, including periodontitis [3,5]. NF-κB receptor activity is also accompanied by other receptors, such as the peroxisome proliferator-activated receptor (PPAR) [7]. The PPAR-γ/c-Fos pathway has been reported to participate in RANKL-induced osteoclastogenesis and the etiopathogenesis of various diseases, including periodontitis, diabetes, and other chronic inflammatory diseases [3].

Furthermore, PPAR-γ agonists were found to be effective in the treatment and prevention of such diseases [3].

PPAR is a nuclear receptor that forms heterodimers with retinoid X receptor (RXR) [8]. PPAR activity is also closely related to the vitamin D receptor (VDR), which also forms heterodimers with RXR [9]. The PPAR and VDR have been suggested to induce autophagy in periodontal tissues and to contribute to the etiopathogenesis of chronic inflammatory diseases such as periodontitis and rheumatoid arthritis [10,11]. PPAR also modulates primary inflammatory cells, especially macrophages, by inducing M2 macrophage maturation and altering the M1/M2 cell ratio [12]. Macrophage cells are known to express significant amounts of PPAR-γ [13].

However, PPAR-γ has two distinct activities; one is anti-inflammatory activity in bacterial infections, via suppression of interleukin (IL)-1β, IL-6, and tumor necrosis factor (TNF)-α [8,14]. The other is the potential modulation of inflammatory cell dynamics through apoptosis-related mechanisms [15]. Reduced chemotactic response and neutrophil migration have also been reported following PPAR-γ activation [16].

Inflammation is the main event that initiates periodontitis, and the severity of inflammation, as well as the cells and biochemical processes involved in inflammatory reactions in periodontal tissues, are crucial for disease development and progression [17]. Previous studies have reported altered expression of nuclear receptors such as PPAR-γ, RXR-α, and VDR in peri-implant inflammatory lesions [18]. However, the role of the PPAR-γ, RXR, and VDR in the etiopathogenesis of periodontitis and the expression levels in the diseased and healthy gingiva have not been determined yet. Therefore, the present study aimed to compare the expression of PPAR-γ, RXR-α, and VDR between grade B and grade C periodontitis with the same stage involvement (stage 3), while using periodontally healthy gingiva as a reference group. Identifying biological correlates of periodontitis grade may improve risk stratification and support translational host-modulatory strategies; therefore, defining grade-associated expression profiles of PPAR-γ, RXR-α, and VDR may have clinical value.

## 2. Materials and Methods

This is a cross-sectional clinical study performed on gingival biopsies obtained from healthy volunteers and periodontitis patients. The study was conducted in accordance with the Declaration of Helsinki, and the protocol was approved by the Ethics Committee of Tokat Gaziosmanpasa University (19-KAEK-191) in July 2019. The study was performed at Tokat Gaziosmanpaşa University Faculty of Dentistry Department of Periodontology. All participants were informed regarding the research, and written informed consent was obtained. After a detailed oral and radiologic examination, healthy volunteers and periodontitis patients were assigned to the relevant study groups.

Three study groups were created, and 45 participants were enrolled in the present study.

The study groups were as follows:

Group 1: Healthy individuals (mean age 42.34 ± 3.89, 9 women, 6 men);

Group 2: Periodontitis patients-stage 3 grade B, (mean age 43.12 ± 3.16, 10 women, 5 men);

Group 3: Periodontitis patients-stage 3 grade C, (mean age 42.78 ± 2.99, 10 women, 5 men).

The age and gender distribution among the healthy volunteers and periodontitis patients were similar. The inclusion criteria were systemic health, no drug use, no previous periodontal treatment within six months, and no tobacco use. Participants with any systemic health condition, disease, pregnancy, lactation, or menopause were excluded from the study. Patients who used antibiotics within the previous six months were also excluded.

All participants were selected from the database of the Department of Periodontology, Faculty of Dentistry, Tokat Gaziosmanpasa University. Patients with at least five years of clinical follow-up, together with available radiographic and anamnesis records, were evaluated. Periodontitis patients were diagnosed according to the 2017 World Workshop classification of periodontal and peri-implant diseases and conditions. Although longitudinal records were available, grade determination in the present study was based primarily on the percentage of radiographic bone loss relative to age [4]. This approach was preferred to minimize potential bias associated with repeated bone loss measurements on panoramic radiographs. All clinical and radiographic evaluations were performed by an experienced clinician. To improve consistency in grading, patient assessments were repeated at two different time points, and only cases with consistent diagnostic outcomes were included in the study, thereby minimizing potential misclassification.

### 2.1. Clinical Parameters

Plaque index (PI) [19], gingival index (GI) [20], and clinical attachment loss (CAL) were determined as full-mouth clinical periodontal measurements. Measurements were performed from three points on mesial, middle, and distal regions on both buccal and palatal aspects. CAL levels were measured as the distance in millimeters from the cementoenamel junction to the bottom of the periodontal pocket, and a Williams-type periodontal probe (Hu-Friedy Co., Chicago, IL, USA) was utilized. The gingiva around teeth with probing depths and attachment loss exceeding 6 mm was selected for gingival sampling in periodontitis patients. For healthy volunteers, teeth with a probing depth of 3 mm without attachment loss were chosen.

### 2.2. Gingival Sampling

Gingival biopsies were obtained using a standardized gingivectomy-based sampling protocol in all groups, similar to our previous histological studies [21,22]. In healthy individuals, samples were collected from sites indicated for crown-lengthening or gingivectomy procedures and showing no clinical signs of inflammation. In periodontitis patients, samples were obtained during periodontal therapy from inflamed gingival areas with impaired gingival topography. In all groups, biopsies were collected from maxillary posterior teeth by the same clinician. Following local anesthesia, a standardized 2 × 2 mm gingival tissue sample was resected using a no. 11 blade. The collected samples were immediately fixed in 10% neutral buffered formalin for 24–48 h prior to histological processing. Efforts were made to standardize biopsy depth and tissue dimensions under clinical conditions.

### 2.3. Histopathological Evaluation

All tissue samples were washed under tap water to remove formalin and dehydrated with an alcohol series. Afterward, samples were cleared with xylene and embedded in paraffin. Then, 5 µm serial sections were cut from the paraffin blocks. Hematoxylin and eosin (H&E) staining and immunohistochemistry were performed. All histological procedures and evaluations were performed by an experienced researcher who was blinded to the clinical group allocation of the samples. Connective tissues were evaluated, and inflammatory cell infiltration and fibroblast cells were counted in the H&E-stained slides using a light microscope (Nikon Eclipse, E 600, Tokyo, Japan). For cell counting, connective tissue neighboring the gingival epithelium was marked, and a cell counting frame of 10,000 µm^2^ was marked. Cell counting was performed under 1000× magnification. The fibroblasts and inflammatory cells (neutrophils, lymphocytes, eosinophils, and macrophages) within the frame were counted. The measurements were performed from three different points, and the mean value of these three measurements was recorded.

### 2.4. PPAR-γ, RXR-α, and VDR Immunohistochemistry

Three slides were selected from each paraffin block and underwent the immunohistochemistry procedure for each parameter. Firstly, samples were deparaffinized with xylene and underwent an ethanol series for dehydration. Then, antigen retrieval was performed (10 mM sodium citrate buffer (pH 6.0) for two h at 70 °C), and sections were subjected with 3% hydrogen peroxide to suppress endogenous peroxidase activity. After washing thrice for 5 min with phosphate-buffered saline solution (PBS) (3 × 5), sections were incubated with normal rabbit serum for 30 min. Then, primary antibody incubation was performed overnight with goat polyclonal anti-PPAR-γ antibody (1:250, Elabscience, Wuhan, China), anti-RXR-α antibody (1:250, Elabscience, Wuhan, China), and anti-VDR antibody (1:250, Elabscience, Wuhan, China). Sections were washed 3 × 5 with PBS and subjected to a streptavidin–horseradish peroxidase-conjugated reagent for 30 min. After 3 × 5 PBS washes, sections were incubated with Aminoethyl carbazole as a chromogen to visualize immunoreactivity. After washing again with 3 × 5 PBS, slides were counterstained with Gill’s hematoxylin and evaluated under light microscopy (Nikon Eclipse, E 600, Tokyo, Japan) [23,24].

### 2.5. H Score Analysis

IHC evaluation was performed for each marker under 400× magnification using a light microscope. All evaluations were performed by an experienced researcher who was blinded to the samples and study groups. For each patient and each marker, three consecutive histological sections were selected. In each section, three different regions of interest (ROIs) located in the connective tissue adjacent to the epithelium were evaluated using a counting frame of 10,000 µm^2^. Thus, a total of nine measurements were obtained for each marker in each patient. All cells within the selected ROI were scored according to staining intensity as unstained, slightly stained, moderately stained, or densely stained. H-scores were calculated for each ROI using the formula ∑Pi(i + 1), where i represents staining intensity, and Pi represents the percentage of stained cells [23,24]. The mean H-score value obtained from the nine measurements was used for statistical analysis for each receptor in each patient.

### 2.6. Statistical Analysis

The power of the study was calculated based on another study with a similar design, and the power was calculated as 85% (Balci Yuce et al. 2019 [23]). IBM SPSS Statistics (IBM Corp., Armonk, NY, USA) (vs. 20.00) was used for statistical analysis. All data were presented as mean ± SD. Normality was assessed using the Shapiro–Wilk test, and homogeneity of variances was checked with Levene’s test. Group comparisons were performed using one-way ANOVA followed by Tukey’s post hoc test. *p* < 0.05 was considered statistically significant.

## 3. Results

The age, PI, GI, and CAL values of the study groups are presented in Table 1. PI, GI, and CAL values were lower in the healthy group than in the other groups (*p* < 0.05).

### 3.1. Histopathological Evaluation Results

**Figure 1:**
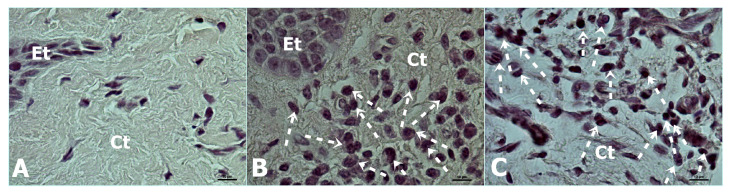
Representative histological images of the study groups. (**A**) Healthy control group, (**B**) periodontitis patients—stage 3 grade B, and (**C**) periodontitis patients—stage 3 grade C. Interrupted white arrows indicate inflammatory cells. Ct: connective tissue and Et: epithelial tissue.

**Figure 2:**
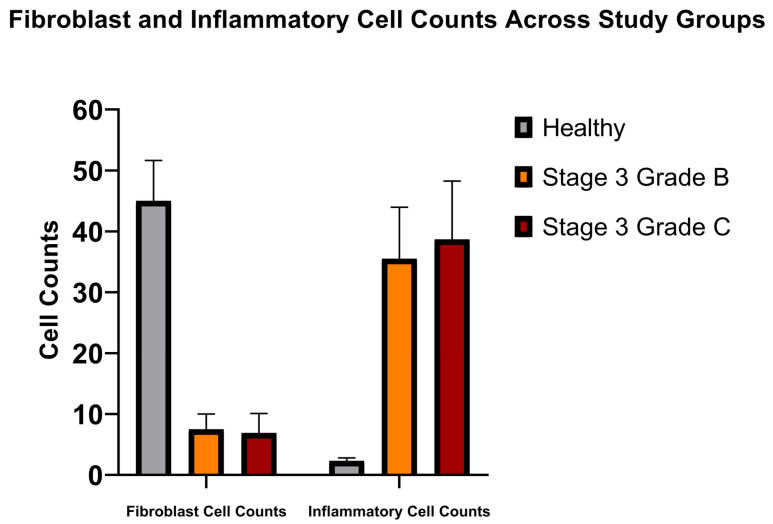
Graphic demonstration of fibroblast and inflammatory cell counts in the study groups. Data are presented as mean ± SD.

The connective tissue in healthy volunteers contained more fibroblast cells and fewer inflammatory cells, whereas the periodontitis groups showed dense inflammatory cell infiltration. Fibroblast cells were also lower in the periodontitis groups. Healthy volunteers had higher fibroblast cell density compared to the other groups (*p* < 0.05) (Figure 1 and Figure 2, Table 2). The periodontitis patients with stage 3 grade B and C had similarly lower fibroblast cells (*p* > 0.05). As for the inflammatory cells, the healthy group had significantly lower inflammatory cells compared to the other groups, and the cell counts in the periodontitis patients were similar (*p* > 0.05) (Figure 1 and Figure 2, Table 2). All inflammatory cell types were identified in the sections individually, but they were all recorded under one heading.

### 3.2. PPAR-γ, RXR-α, and VDR Immunohistochemistry Results

The healthy control exhibited significantly lower PPAR-γ expression than the periodontitis groups (*p* < 0.05). Periodontitis patients with stage 3 grade B also had lower PPAR-γ expression than those with stage 3 grade C (*p* < 0.05).

RXR-α levels of healthy controls were significantly higher than those of the periodontitis groups (*p* < 0.05). Both groups of periodontitis had similarly lower levels of RXR-α (*p* > 0.05).

**Figure 3:**
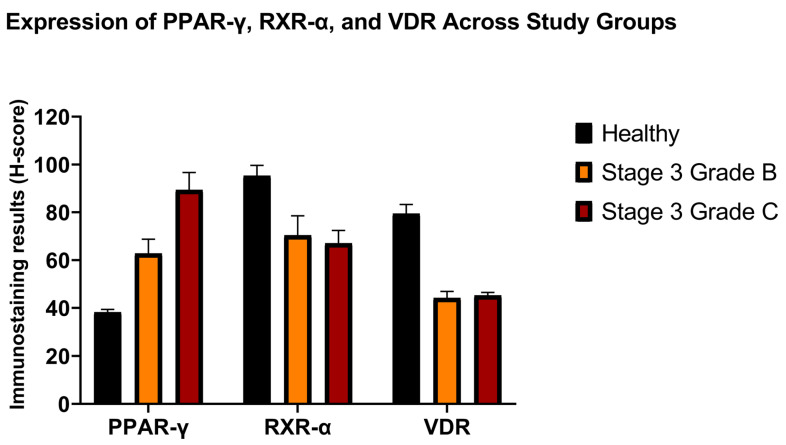
Quantitative immunohistochemical expression of PPAR-γ, RXR-α, and VDR in gingival tissues from healthy controls and periodontitis patients (stage 3 grade B and grade C). Expression levels were evaluated using the H-score method. Data are presented as mean ± SD.

**Figure 4:**
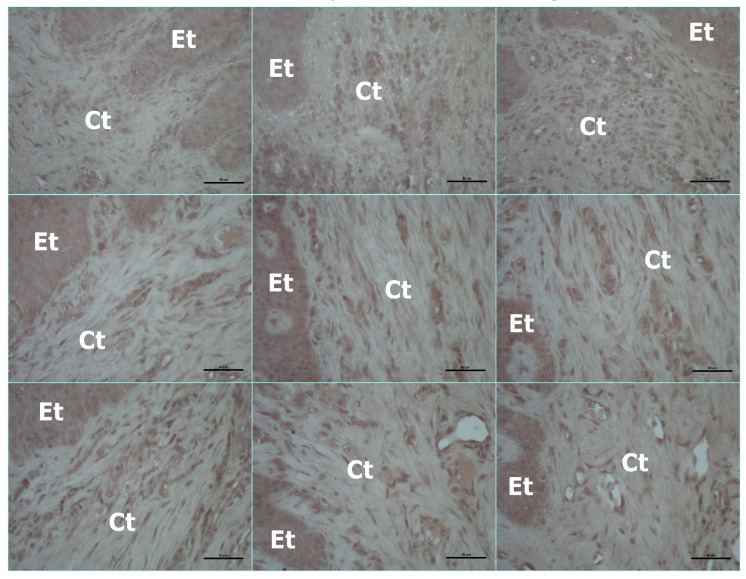
Representative immunohistochemical staining of PPAR-γ, RXR-α, and VDR in gingival tissues from healthy controls and periodontitis patients (stage 3 grade B and grade C). Columns represent study groups (**left** to **right**): healthy control, stage 3 grade B periodontitis, and stage 3 grade C periodontitis. Rows represent immunostaining for PPAR-γ (**top**), RXR-α (**middle**), and VDR (**bottom**). Brown staining indicates positive immunoreactivity. Et: epithelium; Ct: connective tissue. Scale bar: 50 µm.

Similarly, VDR levels of the control group were also higher than those of the periodontitis groups (*p* < 0.05). The immunohistochemistry results were presented in Table 2, as well as Figure 3 and Figure 4 (*p* < 0.05).

## 4. Discussion

The present study evaluated the PPAR-γ, RXR-α, and VDR expression in the healthy gingiva and gingival samples of patients diagnosed with either periodontitis with stage 3 grade B or grade C. The results revealed that PPAR-γ increased in the diseased gingival tissues, and the increase was more evident in the periodontitis group with stage 3 grade C. RXR-α and VDR expressions decreased in the periodontitis group, regardless of the grade of the disease. Furthermore, the gingival tissues of the periodontitis patients exhibited lower fibroblast cell counts and higher inflammatory cell counts, irrespective of the grade of the disease, compared to the control group. Taken together, these findings suggest that grade-related clinical aggressiveness may be accompanied by increased PPAR-γ expression and a shift in cellular composition within gingival connective tissue, whereas RXR-α and VDR changes appear to be more closely related to periodontal disease presence than to grade differences.

Periodontitis causes persistent low-grade inflammation and alterations in gingival tissue composition [3,17]. In periodontal tissues, increased collagenase activity, reduced cell proliferation, and enhanced apoptosis and autophagy have been reported to accompany inflammation. NF-κB signaling triggers downstream inflammatory reactions, which can impair fibroblast proliferation and differentiation [25]. The fibroblast cell density tends to increase in severe inflammation, while inflammatory cell infiltration increases [17,26]. Apart from factors that reduce fibroblast proliferation and differentiation, apoptosis and autophagy may also contribute to reduced fibroblast cell counts in periodontal tissues [27,28]. Inflammation alters tissue homeostasis both directly and indirectly through apoptosis- and autophagy-related pathways [28,29]. The cell composition of the connective tissue in gingival diseases consists of a dense inflammatory infiltrate, resulting from up-regulated inflammatory pathways [5]. In the present results, diseased gingiva exhibited lower fibroblast and higher inflammatory cell counts than healthy gingiva. In addition, different grade involvement of periodontitis caused a similarly low fibroblast cell density. The inflammatory cell counts, on the other hand, were significantly higher in both periodontitis groups, while they were lowest in the healthy controls. Furthermore, the nuclear receptors PPAR-γ and VDR were found to be related to up-regulated autophagy and participate in the development of chronic destructive inflammatory diseases such as rheumatoid arthritis [11]. Therefore, PPAR-γ and VDR levels in the diseased gingival tissues might be related to the reduced fibroblast cell density in periodontal inflammation. Nonetheless, the inflammatory reaction to bacterial challenge leads to marked inflammatory cell accumulation in gingival tissues, resulting in a reduction in the regular structural components of healthy tissue [30]. Overall, these observations support the view that the cellular shift we observed (reduced fibroblast density and increased inflammatory infiltrate) represents a core tissue-level feature of periodontitis, and that the altered PPAR-γ/VDR signaling profile detected in our samples may reflect disease-associated regulatory changes rather than a simple bystander effect.

PPAR-γ plays a significant role in inflammation, primarily through its regulatory effects on inflammatory cells [13]. In addition, PPAR-γ has been associated with apoptosis-related regulation of inflammatory cells and with modulation of inflammatory responses [15], potentially through activation of the AMP-activated protein kinase signaling pathway [31]. Reduced neutrophil migration and chemotactic response have also been reported following PPAR-γ activation [16]. Moreover, its role in soft and hard tissue destruction may be associated with RANKL-mediated pathways [3,32]. Consistent with these anti-inflammatory properties, pharmacological activation of PPAR-γ has been shown to improve inflammation and inflammation-driven tissue destruction [3].

In the present study, PPAR-γ expression was significantly higher in grade C periodontitis compared with the other groups, suggesting altered inflammatory regulation in more severe disease. Although PPAR-γ is known for its anti-inflammatory properties, the increased expression observed in grade C periodontitis should be interpreted cautiously within the observational design of the present study. Rather than indicating reduced inflammatory activity, higher PPAR-γ expression may be associated with altered inflammatory regulation in diseased gingival tissues.

However, the anti-inflammatory activity of PPAR-γ has also been reported [8,14]. Accordingly, PPAR-γ agonists were found to suppress IL-6, IL-8, inducible nitric oxide synthase, and COX-2 levels [31]. Decreased biofilm formation of *Porphyromonas gingivalis* and NF-κB activation was also demonstrated in response to PPAR-γ agonist treatment [33]. Previous studies have reported that inflammatory conditions may be associated with altered PPAR-γ expression. However, the direction and magnitude of this relationship appear to depend on the tissue context and stage of the inflammatory response [3,34]. These apparently divergent observations may reflect the context-dependent nature of PPAR-γ signaling, which can vary according to tissue microenvironment and stage of the inflammatory response. Borges et al. recently reported that PPAR-γ levels decreased when the VDR levels increased in vivo [35]. VDR is the receptor for vitamin D, and reduced vitamin D levels were associated with decreased VDR levels and increased PPAR-γ levels [36]. The levels of PPAR-γ in the present study were lowest in the healthy group. Furthermore, periodontitis patients with grade C involvement had significantly higher levels of PPAR-γ compared to periodontitis patients with grade B involvement. Given the increased inflammatory cell infiltration observed in the diseased groups, the higher PPAR-γ expression detected in our samples may be associated with the overall inflammatory cell response within the gingival tissues. Since inflammatory cells were evaluated collectively in the present study, cell-specific interpretations should be made with caution.

As for VDR expression, healthy controls had significantly higher levels than periodontitis patients, and VDR levels were not affected by the grade of involvement of periodontitis. This pattern is in line with recent literature suggesting an association between impaired vitamin D/VDR-related pathways and periodontal disease [37]. These patterns are consistent with the inflammatory role of PPAR-γ, as evidenced by the increased expression in diseased samples, whereas VDR levels were inversely associated with periodontal disease. PPAR-γ is a strong modulator of inflammation through its association with inflammatory cell activity and RANKL-related pathways [18]. In addition, VDR has been shown to inhibit NF-kB activation through IkB-α and exhibit a potent inhibitory feature to inflammation [38]. In this context, the combination of elevated PPAR-γ and reduced VDR expression in our periodontitis samples provides a possible biological context for inflammatory alterations in gingival tissues. However, only PPAR-γ differed between grade B and grade C periodontitis.

RXR is a nuclear receptor that serves as an obligatory partner for several nuclear receptors, including PPAR-γ and VDR, thereby influencing their transcriptional activity. Furthermore, RXRα is widely expressed in osteoblast and osteoclast lineage cells and has been reported to modulate the differentiation and function of osteoblast and osteoclast cells. RXR agonists also increased bone turnover, and the role of RXR in the inhibition of apoptosis and inflammation was also demonstrated [39,40,41,42]. The most significant clinical relevance lies in the receptor RXR binds to, either PPARγ or VDR, and determines the course of the inflammatory status [43]. Therefore, PPAR-γ, RXR, and VDR levels were evaluated together in this study. VDR and RXR heterodimerization plays a crucial role in cancer prevention and has been reported to provide significant anti-inflammatory activity by suppressing the NF-κB pathway and COX production [44,45]. The present results also showed decreased RXR levels along with VDR in the periodontitis samples. Similarly to the VDR results, grade involvement did not affect RXR expression either.

Taken together, the present findings suggest that grade-related variation in stage 3 periodontitis is mainly reflected by PPAR-γ expression. In contrast, RXR-α and VDR appear to be more closely associated with the presence of periodontal disease itself than with differences in grade. Because of the cross-sectional and observational nature of the present study, the findings should be interpreted as associations rather than direct evidence of underlying molecular mechanisms.

This study has limitations: the cross-sectional design and modest single-center sample limit causal inference and generalizability. Although the biopsy technique, anatomical region, operator, and sample dimensions were standardized across groups, differences in clinical context between healthy controls and periodontitis patients may still have influenced local tissue responses. Another limitation of the present study is the absence of bleeding on probing (BOP) measurements, which may provide additional information regarding the current inflammatory status. Additionally, the absence of formal intra- and inter-examiner reliability analyses for histological and immunohistochemical assessments should be considered as a limitation. Further longitudinal studies with quantitative molecular validation are required to clarify the translational value of the grade-associated difference observed for PPAR-γ and the disease-associated changes observed for RXR-α and VDR.

## 5. Conclusions

The present study demonstrated distinct nuclear receptor expression patterns in gingival tissues from periodontally healthy individuals and from patients with stage 3 periodontitis of grade B and grade C. Higher PPAR-γ expression in grade C periodontitis suggests a grade-related difference in host-response regulation within the same disease stage. In contrast, RXR-α and VDR expression primarily distinguished healthy from diseased gingival tissues rather than grade B from grade C periodontitis. However, these findings should be interpreted considering the cross-sectional design and the semi-quantitative nature of immunohistochemistry. Future studies incorporating longitudinal designs and confirmatory molecular assays (e.g., Western blot, RT-PCR, or ELISA) are warranted to clarify clinical relevance and translational potential.

## Figures and Tables

**Table 1:** Mean age, plaque index, gingival index, and clinical attachment loss of the study groups. Data are presented as mean ± SD. * *p* < 0.05 vs. healthy controls.

Groups/ Parameters	Healthy Controls	Stage 3 Grade B	Stage 3 Grade C
Plaque index	0.50 ± 0.50	2.50 ± 0.20 *	2.55 ± 0.30 *
Gingival index	0.50 ± 0.50	2.25 ± 0.50 *	2.50 ± 0.55 *
Clinical attachment loss	1.50 ± 0.50	6.25 ± 0.40 *	6.40 ± 0.90 *
Age	42.34 ± 3.89	43.12 ± 3.16	42.78 ± 2.99

**Table 2:** Immunohistochemical expression levels of PPAR-γ, RXR-α, and VDR, and fibroblast and inflammatory cell counts in the study groups. Data are presented as mean ± SD. * *p* < 0.05 vs. healthy controls, ^†^ *p* < 0.05 vs. stage 3 grade B periodontitis group.

Groups/ Parameters	Healthy	Stage 3 Grade B	Stage 3 Grade C
Fibroblast counts	45.20 ± 5.40	7.50 ± 2.50 *	6.90 ± 3.20 *
Inflammatory cell counts	2.30 ± 0.50	35.50 ± 8.45 *	38.69 ± 9.56 *
PPAR-γ	38.20 ± 1.19	62.75 ± 5.99 *	89.34 ± 7.28 *^†^
RXR-α	95.30 ± 4.34	70.41 ± 8.12 *	67.02 ± 5.37 *
VDR	79.44 ± 3.81	44.18 ± 2.72 *	45.26 ± 1.22 *

## Data Availability

The data presented in this study are available from the corresponding author upon reasonable request.
